# Heterogeneous colonic content: A prenatal sonographic manifestation of lysinuric protein intolerance

**DOI:** 10.1002/ccr3.2780

**Published:** 2020-03-06

**Authors:** Christos Dimitriou, Souha Saliba, Xavier Peyrassol, Wafa Ben Abbou, Marie Cécile Nassogne, Carine Neugroschl, Elsa Wiame, Anne De Leener, Marie Cassart

**Affiliations:** ^1^ Department of Radiology Iris Hospitals South Brussels Belgium; ^2^ Molecular Genetic Laboratory ULB Erasme Brussels Belgium; ^3^ Department of Fetal Medicine Iris Hospitals South Brussels Belgium; ^4^ Department of Pediatric Neurology and hereditary metabolic diseases CHU Saint‐ Luc Brussels Belgium; ^5^ Laboratory of Biochemistry de Duve Institute Brussels Belgium; ^6^ Department of Genetics CHU Saint‐Luc Brussels Belgium

**Keywords:** enterolithiasis, metabolic disease, prenatal diagnosis

## Abstract

We report a fetus with heterogeneous colonic content, an isolated sonographic prenatal sign of lysinuric protein intolerance, a very rare metabolic disease. Familial genetic enquiries confirmed heterozygote mutation in the implicated gene in parents. The prenatal diagnosis led to neonatal dietary adaptation and avoided acute complications.

## Graphical abstract

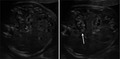

## 1 INTRODUCTION

Heterogeneous colonic content in prenatal ultrasound (US) is firstly suggestive of enterolithiasis. They result from meconium ball‐like precipitation subsequently to urine impregnation. Such an aspect should always alert the sonographer to depict signs of anorectal malformations with fistula between the digestive and urinary tracts. When such a diagnosis is excluded, further rarer etiologies should be suggested.

We report a case of a fetus with prenatal diagnosis of a rare hereditary metabolic disease for which the only signs on prenatal US were the abnormal colonic content.

## 2 CASE REPORT

A 25‐year‐old, gravida 1, para 0, Aramaic woman without significant medical history was referred to our fetal medicine center for further evaluation because a routine prenatal US performed at 26 weeks of gestation revealed isolated hyperperistaltic colon with heterogeneous content.

**Figure 1:**
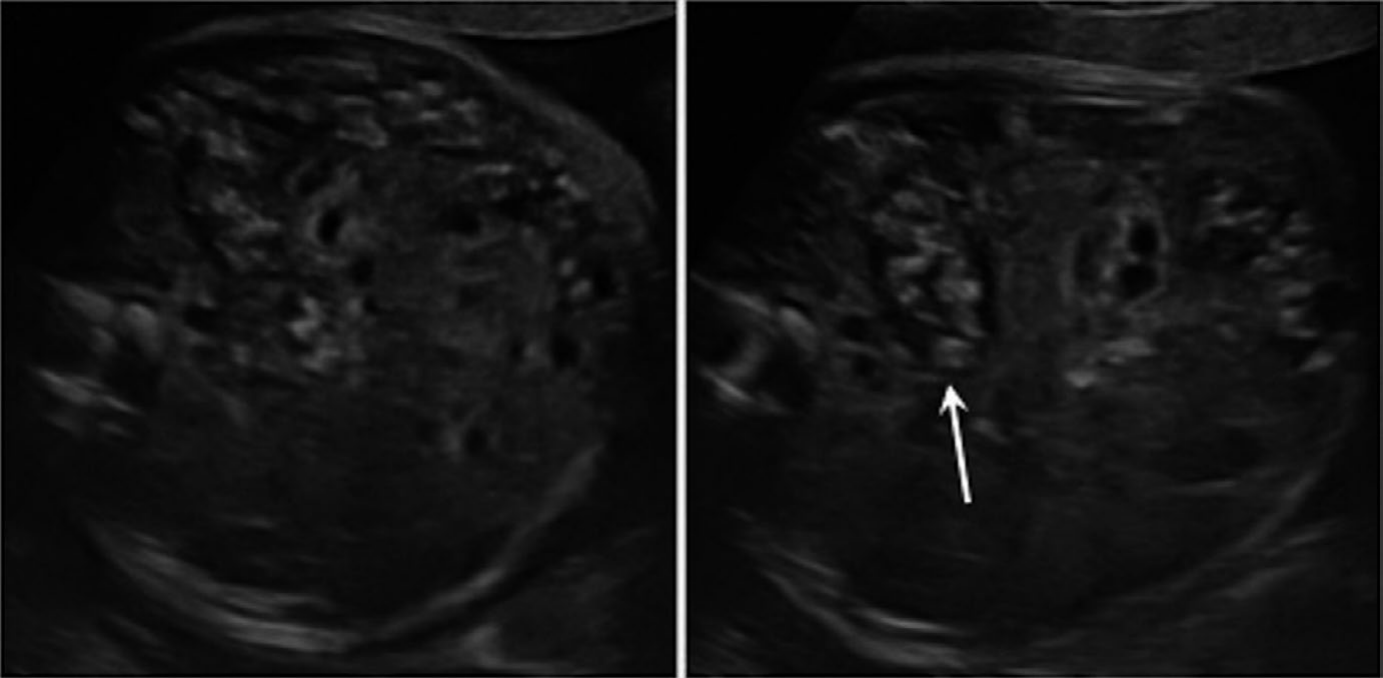
Abdominal axial US slices on the abdomen of the 26‐wk‐old fetus presenting heterogeneous colonic content (arrow) without dilatation or ascites

The sonographic examination showed a normal female fetus with biometry and amniotic fluid within normal ranges for gestational age (GA). It also depicted multiple hyperechoic ball‐like structures rolling within the bowel (Figure 1). The intestinal wall presented a normal aspect. The fetus had no ascites, and no intestinal dilatation or extraluminal calcified meconium. Based on the abnormal colonic content, diagnosis of anorectal malformation (ARM) with urodigestive fistula, cystic fibrosis, cystinuria, or a fetal infection was mentioned. The follow‐up US examinations showed the persistence of echogenic foci within the colon and rectum. The parents did not accept amniocentesis for genetic testing. There was no documented evidence of a recent maternal seroconversion to any viral infection (CMV, EBV, parvovirus, or herpes simplex virus). Parental screening for frequent mutations involved in cystic fibrosis was also negative (exclusion of 35 mutations in the CFTR gene by the Inno‐Lipa^®^ kit).

**Figure 2:**
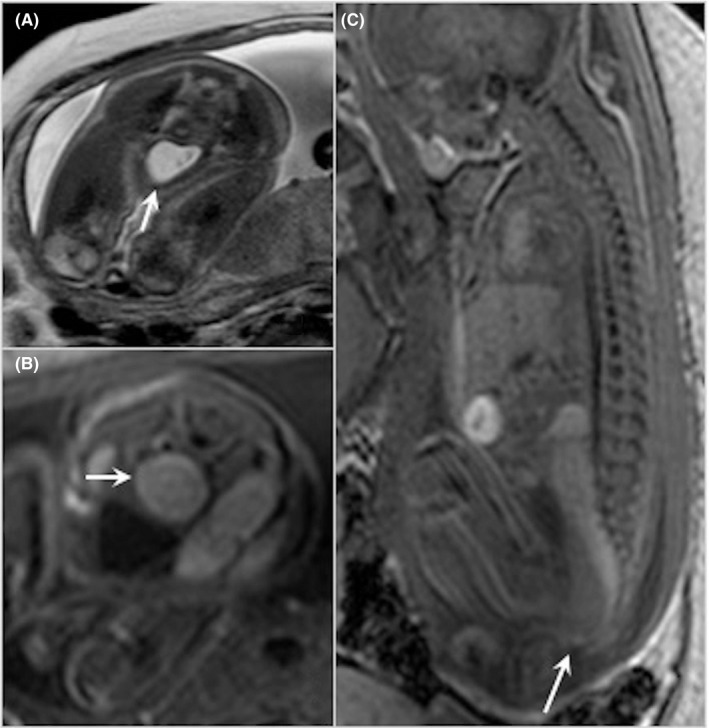
Fetal MRI performed at 31 wk of GA. A, axial T2‐weighted image showing a normal hypersignal bladder (arrow); B, axial T1‐weighted image showing the normal hypersignal rectosigmoid loop (arrow); C, sagittal T1‐weighted image depicting the normal distal end of the rectum to the anal line (arrow)

A magnetic resonance imaging (MRI) was performed at 31 weeks of GA to rule out anorectal malformation. It showed a normal aspect of the upper digestive tract. The colon and rectum were not dilated and showed intermediate signal on T2 sequence and a high signal intensity on T1 sequence compatible with normal meconial content. The rectum was ending on the anal line normally positioned under the bladder neck, thereby reasonably excluding the hypothesis of high ARM with urodigestive fistula (Figure 2).

Genetic counseling revealed a familial history of young cousin known for being affected by a very rare metabolic disease, called lysinuric protein intolerance (LPI). This child was born nine years ago from a consanguineous marriage. Prenatal US had also revealed colon with heterogeneous content without definitive diagnosis. This girl presented in the neonatal period with hyperammonemia. She is now 9 years old with growth −3 SD below the normal curves and a mild language delay. She attends a normal school and benefits from speech therapy. She receives a diet with mild protein restriction and carnitine and citrulline complementation. Hematologic findings include thrombocytopenia, increased plasma concentrations of triglycerides, ferritin, and LDH, as described in LPI. Metabolic investigations led to the diagnosis of LPI with c.1013G>A (p.G338D) homozygous mutation in exon 8 of the *SLC7A7* gene. This mutation was initially described in a Swedish family.^1^ Although no functional test was carried out, this nucleotide change was not found in a control cohort, and the allelic frequency is extremely low (0.0003979% according to gnom AD). In all the families, affected children were homozygous carriers and both parents were shown healthy heterozygous carriers. Therefore, this change is considered as probably pathogenic (class IV).^2^

**Figure 3:**
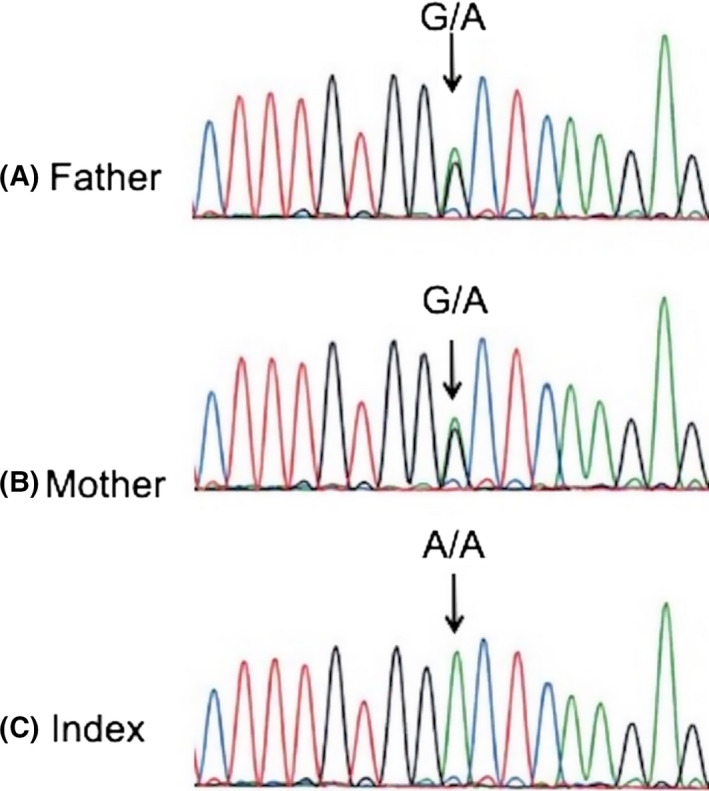
Specific region of the exon 8 of SLC7A7 gene was amplified and sequenced by Sanger sequencing. Both parents are heterozygous carriers of the G>A change at the 1013 nucleotide level. c.1013G>A (A,B) leading to p.G338D mutation. The child index case was shown homozygous for the mutation (C)

The molecular analysis performed by Sanger sequencing on the parents of the actual pregnancy at 31 weeks of pregnancy revealed that they were heterozygous for c.1013G>A (p.G338D) familial mutation of the *SLC7A7* gene (Figure 3).

The baby was born at 36 weeks and 4 days after induction of labor because of premature rupture of the membranes. The newborn weighed 2380 g. Chromatography of amino acids showed a mild increased level of glutamine in plasma and an increased urinary excretion of arginine, ornithine, lysine, and orotic acid. Molecular analysis confirmed that the baby was homozygous for the c.1013G>A (p.G338D) mutation in exon 8 of the *SLC7A7* gene mutation (Figure 3). The diagnosis of LPI was then established allowing a precocious medical management consisting in immediate dietary adaptation with mild protein restriction and carnitine and citrulline complementation. She is now 3.5 years old with growth in the lower part of the normal curves and a normal psychomotor development.

## 3 DISCUSSION

Lysinuric protein intolerance is a rare autosomal recessive metabolic disease with defective transport, at the basolateral membrane of epithelial cells in the kidney and intestine, of cationic amino acids (CAAs) including arginine, lysine, and ornithine. LPI is caused by biallelic pathogenic variants in the *SLC7A7* gene that encodes the y + LAT‐1 protein (light subunit of a CAA transporter—heavy subunit is 4F2hc, encoded by the *SLC3A2* gene).^1^ Fifty mutations have been currently reported, initially in the Finnish population (where the prevalence is 1/60 000). LPI seems also frequent in the Italian population and has been sporadically reported worldwide.^3^, ^4^, ^5^

This metabolic disease presents variable symptoms, including vomiting after weaning, failure to thrive, hepatosplenomegaly, osteoporosis causing easy bone fractures, muscular hypotonia, and hypotrophy. Mental retardation, altered immune response, and chronic renal disease are also reported in the spectrum of complications. Life‐threatening episodes of coma due to metabolic imbalance provoked by hyperammonemia and severe pulmonary complications may lead to respiratory insufficiency and death.^6^

The main biochemical findings are decreased CAA intestinal absorption, increased CAA renal excretion, and high orotic aciduria.^3^ Therefore, the postnatal diagnosis is based on the chromatography of both plasma and urine amino acids that shows a reduction in plasmatic levels of arginine, ornithine, and lysine and an increased urinary excretion of the same AA.

The clinical symptoms of this disease could be limited or managed by excluding CAA from nutrition.

It is recommended to the patients to have a low‐diet protein to avoid the hyperammonemia with citrulline supplement. Lysine supplementation can be given to decrease the pulmonary complications, and ornithine and citrulline supplementation can be given to restore the urea cycle.^6^ In the cases of alveolar proteinosis, it is recommended to perform a bronchoalveolar wash.^7^

The prognosis of LPI depends on the age at diagnosis and on the pulmonary complications.

A prenatal diagnosis, by precocious adaptation of the diet, will allow limitation of the symptoms due to the metabolic imbalance and avoid early‐life complications.

The prenatal diagnosis of this rare metabolic hereditary disease can be made on genetic analysis in families with previous medical history of affected children, but to our knowledge, none has been suspected on abnormal sonographic findings.^8^

Prenatal diagnosis of hyperechoic *small bowel* discovered earlier in pregnancy (second trimester) is quite frequent. It can be related to aneuploidy (T21), mesenteric ischemia subsequently to hemodynamic redistribution in growth‐retarded fetuses, intra‐amniotic hemorrhage, cystic fibrosis, or congenital infection.^9^ Our fetus presents heterogeneous *colonic content*, which is a very different context with significant clinical impact. Such heterogeneous colonic content should raise the suspicion of enterolithiasis due to urodigestive fistula at first, abnormal meconial structure in case of cystic fibrosis at second, if both are excluded, rarer metabolic diseases should be considered.^10^, ^11^, ^12^

Fetal MRI is a good complementary tool to US in such a context. It allows a good characterization of digestive tract content differentiating small bowel filled with amniotic fluid (hyperintense on T2‐weighted sequences) from colon filled with meconium (hyperintense on T1‐weighted sequences). The different colonic content (suspected on US aspect) in our case of LPI did not modify the signal on MRI and did not help in establishing the diagnosis. However, the normal colonic content signal, excluding the presence of urine, associated with a normal anatomical position of the rectal pouch distally to the bladder neck allowed us to exclude high ARM with fistula.^13^ Congenital cystinuria has also been evoked, but it typically presents with homogeneous highly hyperechoic colonic content on US and normal T1 and T2 signal of this content on fetal MRI.^14^, ^15^ Cystic fibrosis has been excluded on genetic testing. Rarer metabolic diseases were then searched by the geneticist who identified, in her family enquiry, a cousin of the baby affected by LPI and oriented the genetic researches as described above. The mutations involved in LPI were then depicted in both parents and led to the correct prenatal diagnosis. The prenatal diagnosis allowed better management of the newborn avoiding life‐threatening medical complications.

This case report outlines the importance of a teamwork collaboration integrating different specialties (obstetricians, radiologists, geneticists, and pediatricians) working together for the best care of fetus, newborns, and families.

## 4 CONCLUSION

The prenatal heterogeneous colonic content without any signs of ARM should include the differential diagnosis of metabolic diseases and suspect recurrence in case of known familial history. However, it is a single observation that should be confirmed by other cases in order to generalize the described prenatal sonographic pattern associated with this rare metabolic disease.

The advantage of prenatal diagnosis is the immediate neonatal dietary adaptation that avoids early‐life complications of the disease and allows better management of the newborn.

## CONFLICT OF INTEREST

None declared.

## AUTHOR CONTRIBUTION

Drs Dimitriou and Saliba: collected data and wrote the case report. Drs Ben Abbou: performed prenatal US. Drs Nassogne: performed medical follow‐up of the affected children. Drs Peyrassol, Wiame, and De leener: performed the genetic diagnosis and analysis in the two children (two different laboratories). Dr Cassart and Neugroschl: coordinated the work, revised the paper, and performed the MRI.
